# ShinyGS—a graphical toolkit with a serial of genetic and machine learning models for genomic selection: application, benchmarking, and recommendations

**DOI:** 10.3389/fpls.2024.1480902

**Published:** 2024-12-24

**Authors:** Le Yu, Yifei Dai, Mingjia Zhu, Linjie Guo, Yan Ji, Huan Si, Lirui Cheng, Tao Zhao, Yanjun Zan

**Affiliations:** ^1^ Tobacco Research Institute, Chinese Academy of Agricultural Sciences, Qingdao, China; ^2^ Department of Plant Biology, Swedish University of Agriculture Sciences, Uppsala, Sweden; ^3^ Department of Population and Public Health Sciences, Keck School of Medicine, University of Southern California, Los Angeles, CA, United States; ^4^ College of Ecology, Lanzhou University, Lanzhou, China; ^5^ College of Horticulture, Northwest Agriculture and Forestry University, Yangling, China

**Keywords:** genomic prediction, BLUP, machine learning, breeding, graphical toolkit

## Abstract

Genomic prediction is a powerful approach for improving genetic gain and shortening the breeding cycles in animal and crop breeding programs. A series of statistical and machine learning models has been developed to increase the prediction performance continuously. However, the application of these models requires advanced R programming skills and command-line tools to perform quality control, format input files, and install packages and dependencies, posing challenges for breeders. Here, we present ShinyGS, a stand-alone R Shiny application with a user-friendly interface that allows breeders to perform genomic selection through simple point-and-click actions. This toolkit incorporates 16 methods, including linear models from maximum likelihood and Bayesian framework (BA, BB, BC, BL, and BRR), machine learning models, and a data visualization function. In addition, we benchmarked the performance of all 16 models using multiple populations and traits with varying populations and genetic architecture. Recommendations were given for specific breeding applications. Overall, ShinyGS is a platform-independent software that can be run on all operating systems with a Docker container for quick installation. It is freely available to non-commercial users at Docker Hub (https://hub.docker.com/r/yfd2/ags).

## 1 Introduction

Polygenic traits are influenced by multiple genes, leading to continuously distributed phenotypes, such as plant height, grain yield, and resistance to diseases. Accurate predictions of these traits can help crop and animal breeders develop varieties and breeds with significantly improved agronomic performance to meet the growing food demand (Bali and Singla, 2022). Over the past two decades, genomic selection (GS) has become a popular strategy for animal and plant breeding programs and considerably improved the genetic gain for many crops and animals (Liu et al., 2018; Lozada et al., 2019; Marulanda et al., 2016; Sallam and Smith, 2016; Schefers and Weigel, 2012). Various methods have been developed to improve prediction accuracy and computing efficacy (Habier et al., 2011; Jia and Jannink, 2012; Meuwissen et al., 2001; VanRaden, 2008). However, the application of these models requires advanced R programming skills and command-line tools for performing data quality control, formatting input files, and installing dependencies and packages, posing challenges for many breeders.

To make these advanced genomic prediction methods accessible to breeders without programming skills, we developed ShinyGS—a graphical toolkit with a series of genetic and machine learning models for genomic selection. It includes 16 genomic prediction methods implemented in four packages: ridge regression best linear unbiased prediction (rrBLUP) (Meuwissen et al., 2001), the most widely used method based on linear regression models; deep neural network genomic prediction (DNNGP) (Wang et al., 2023); gradient boosting machine (GBM) (Li et al., 2018); and the BWGS (Breed Wheat Genomic Selection pipeline including several genomic prediction methods) (Charmet et al., 2020) method set. DNNGP is based on a deep multilayered hidden neural network architecture that captures complex non-additive effects (Wang et al., 2023). The GBM method utilizes gradient boosting (Friedman, 2001) and stochastic gradient boosting approaches (Friedman, 2002). The BWGS package includes the genomic best linear unbiased prediction (G-BLUP) (VanRaden, 2008), multiple kernel reproducing kernel Hilbert space (MKRKHS) (De Los Campos et al., 2010), ridge regression (RR) (Whittaker et al., 2000), Bayesian ridge regression (BRR) (De Los Campos et al., 2013), least absolute shrinkage and selection operator (LASSO) (Usai et al., 2009), elastic net (EN) (Zou and Hastie, 2005), Bayesian LASSO (BL) (Park and Casella, 2008), Bayes A (BA) (Meuwissen et al., 2001), Bayes B (BB) (Habier et al., 2011), Bayes C (BC) (George and McCulloch, 1993), reproducing kernel Hilbert space (RKHS) (Gianola and van Kaam, 2008), random forest (RF) (Breiman, 2001), and support vector machine (SVM) (González-Recio et al., 2014; Maenhout et al., 2007) models (**Table 1**). In addition, we performed benchmarking analysis using multiple populations and traits with variable population and genetic architecture to provide recommendations for specific breeding applications. ShinyGS is freely available to non-commercial users at Docker Hub (https://hub.docker.com/r/yfd2/ags). This toolkit can significantly simplify genomic prediction applications, making advanced genomic selection methods more accessible and beneficial to breeders.

**Table 1:** Description of each model.

Type	Methods	Abbreviation	Features of the methods	Suitable genetic architecture	Parameter selection guide	Reference
Linear models	Least absolute shrinkage and selection operator	LASSO	L1 penalty, which adds the absolute value of the coefficients to the loss function. Shrinks some coefficients to zero, effectively performing variable selection by excluding certain features	Additive architecture. Useful when a sparse genetic architecture is expected, as it selects relevant markers and excludes unimportant ones	https://cran.r-project.org/web/packages/glmnet/glmnet.pdf	Usai et al. (2009)
	Ridge regression	RR	L2 penalty, which adds the square of the magnitude of coefficients to the loss function. Shrinks all coefficients equally toward zero but does not set any of them exactly to zero	Additive architecture. Suitable for polygenic traits where many small-effect loci contribute to the phenotype. RR is commonly used in genomic prediction models like RR-BLUP	https://cran.r-project.org/web/packages/glmnet/glmnet.pdf	Whittaker et al. (2000)
	Elastic net	EN	Combination of L1 and L2 penalties, balancing the behaviors of both ridge and LASSO. Shrinks coefficients and can set some to zero, similar to LASSO, but also keeps correlated predictors (markers) together, like ridge. It provides flexibility between ridge’s dense solution and LASSO’s sparse solution	Additive architecture. Useful when there is a mix of small and large effect sizes or when markers are correlated	https://cran.r-project.org/web/packages/glmnet/glmnet.pdf	Zou and Hastie (2005)
Linear mixed model (best linear unbiased prediction)	Genomic best linear unbiased prediction	GBLUP	GBLUP is a version of BLUP adapted for genomic selection by using dense SNP data to create a genomic relationship matrix. This allows it to capture relationships more accurately than traditional BLUP, which relies only on pedigree	Additive architecture. Regulated by many small-effect loci with a normal distribution	https://cran.r-project.org/web/packages/BGLR/BGLR.pdf	VanRaden (2008)
	Ridge regression best linear unbiased prediction	rrBLUP	Unlike GBLUP, which uses the genomic relationship matrix to model genetic similarity without explicitly estimating individual SNP effects. RR-BLUP explicitly estimates individual SNP effects through ridge regression, and the total genetic value for an individual is the sum of these SNP effects	Additive architecture. Similar to RR	https://cran.r-project.org/web/packages/rrBLUP/rrBLUP.pdf	Meuwissen et al. (2001)
Linear mixed model (Bayesian methods)	Bayes A	BA	Assumes that each marker effect follows a normal distribution with a constant variance across markers. Uniform shrinkage across all SNPs was applied, with no selection of markers that may have no effect. Suitable for traits controlled by many small-effect loci, as it treats all markers as contributing similarly to the genetic variance	Additive architecture. Similar to GBLUP	https://cran.r-project.org/web/packages/BGLR/BGLR.pdf	Meuwissen et al. (2001)
	Bayes B	BB	Assumes that each marker effect has a normal distribution with marker-specific variances. Provides stronger shrinkage for markers with small or negligible effects, making it more flexible than BA in handling traits with a mixture of large- and small-effect loci	Additive architecture. Often used for traits where only a subset of markers is expected to have significant effects (traits with major QTLs)	https://cran.r-project.org/web/packages/BGLR/BGLR.pdf	Habier et al. (2011)
	Bayes C	BC	Similar to BB but includes an additional mixture distribution that assigns some marker effects directly to zero. Shrinks small-effect markers strongly, while allowing larger-effect markers to retain their impact	Additive architecture. Preferred for sparse genetic architectures, where only a few loci are expected to have large effects	https://cran.r-project.org/web/packages/BGLR/BGLR.pdf	George and McCulloch (1993)
	Bayesian LASSO	BL	BL assumes a Laplace (double-exponential) distribution for marker effects rather than a normal distribution. Strong shrinkage on small-effect markers, which can lead to an outcome similar to the LASSO (L1 regularization) in a Bayesian framework	Additive architecture. Useful when the underlying genetic architecture is suspected to be sparse, as BL emphasizes sparsity more effectively than BB or BC	https://cran.r-project.org/web/packages/BGLR/BGLR.pdf	Park and Casella (2008)
	Bayesian ridge regression	BRR	BRR is a ridge regression model in the Bayesian framework. It assumes that all marker effects follow a normal distribution with constant variance (similar to BA), meaning that all markers contribute to the prediction. Shrinks all marker effects evenly, without excluding any	Additive architecture. Suitable for polygenic traits, where many loci with small effects are expected to contribute to the trait	https://cran.r-project.org/web/packages/rrBLUP/rrBLUP.pdf	De Los Campos et al. (2013)
Non-linear models	Random forest	RF	An ensemble learning method that builds multiple decision trees and averages their predictions. It captures non-linear relationships and interactions between variables. Handles non-linear relationships and interactions well. Robust to overfitting in moderately sized datasets. Computationally intensive for large datasets	Non-additive architecture. Works well for traits controlled by major loci with large effects. Suitable for architectures with complex, non-linear interactions among loci. May struggle with continuous traits if there is high polygenicity (many small effects)	https://cran.r-project.org/web/packages/randomForest/randomForest.pdf	Breiman (2001)
	Support vector machine	SVM	A classification or regression technique that finds a hyperplane in a high-dimensional space to separate or predict data points, often using kernels to capture non-linear relationships. Effective in high-dimensional spaces. Handles non-linear relationships with appropriate kernel choice. Slow training process for large datasets. Limited scalability and less effective for polygenic traits with many small-effect loci	Non-additive architecture. Works well for simple architectures with few large-effect loci. Effective for cases where non-linear boundaries exist in the data but does not capture complex interactions as well as other methods	http://www.csie.ntu.edu.tw/~cjlin/libsvm	González-Recio et al. (2014); Maenhout et al. (2007)
	Gradient boosting machine	GBM	An ensemble method that builds sequential decision trees, with each tree correcting errors from the previous one. It is highly flexible for non-linear relationships. High predictive accuracy for moderate- to complex-trait architectures. Balances speed and predictive power, especially under limited computational resources. Can be prone to overfitting if the dataset is small. Computationally more expensive than simpler models	Non-additive architecture. Works well for traits with both major and minor loci. Suitable for architectures where interactions and non-linear effects exist but are not extremely complex	https://cran.r-project.org/web/packages/gbm/gbm.pdf	Friedman (2001)
	Reproducing kernel Hilbert space	RKHS	A non-linear, kernel-based method that models complex trait architectures by mapping the genetic markers into a high-dimensional feature space. Captures complex, non-linear relationships and interactions effectively. Flexible with different kernel choices to adapt to various genetic architectures. Computationally demanding, especially for large datasets. Sensitive to kernel and hyperparameter choices	Non-additive architecture. Effective for complex traits with polygenic architectures (many small-effect loci) and moderate non-linear interactions	https://cran.r-project.org/web/packages/BGLR/BGLR.pdf	Gianola and van Kaam (2008)
	Multiple kernel reproducing kernel Hilbert space	MKRKHS	An extension of RKHS that uses multiple kernels to capture a range of genetic architectures, allowing for different levels of genetic interactions and polygenicity. Flexibility to model diverse genetic architectures with varying effect sizes. Captures more complex patterns than single-kernel RKHS. Very computationally intensive. Requires careful tuning of multiple kernels and parameters	Non-additive architecture. Suitable for highly complex architectures with multiple levels of genetic effects, including both major loci and polygenic effects. Ideal for architectures with various degrees of interactions	https://cran.r-project.org/web/packages/BGLR/BGLR.pdf	De Los Campos et al. (2010)
	Deep neural network genomic prediction	DNNGP	Combines deep learning with Gaussian processes to capture complex, non-linear relationships and model uncertainty in predictions. It uses deep learning layers to learn representations and a Gaussian process layer for prediction. Highly flexible and capable of capturing very complex patterns and interactions. Can model both non-linearity and uncertainty in predictions. Computationally very demanding; requires significant resources. Prone to overfitting if not properly regularized, especially on small datasets	Non-additive architecture. Suitable for extremely complex architectures, especially when there are high levels of interactions and non-linear effects among loci. Effective for both major effect loci and highly polygenic architectures	https://github.com/AIBreeding/DNNGP/blob/main/EN-Windows-usermanual.pdf	Wang et al. (2023)

## 2 Materials and methods

### 2.1 Example data

Genetic relationship is one of the most important factors that may affect prediction accuracy of genomic selection. As population structure varies between advanced intercross line or germplasm, we choose two types of population to demonstrate the performance of these models. The first is the Goodman maize diversity panel. This panel was built from whole-genome sequencing data from approximately 300 maize lines, covering major maize varieties across the world (Bukowski et al., 2018). Genotypes were downloaded from https://datacommons.cyverse.org/browse/iplant/home/shared/commons_repo/curated/Qi_Sun_Zea_mays_haplotype_map_2018/hmp321_unimputed. We downloaded phenotype records for days to anthesis (DTA), plant height (PH), and ear weight (EW) from Panzea (traitMatrix_maize282NAM_v15-130212.txt) with 282 observations.

For the maize CUBIC population, all 1,404 lines were resequenced. Genotype data were available for download from Liu et al. (2020) (The raw fastq files were uploaded to NCBI SRA with ID as PRJNA597703 and called SNP data in PLINK format available at https://pan.baidu.com/s/1AsPJLTe–gU5EN8aFTMYPA). We downloaded phenotype records for DTA, PH, and EW from Liu et al. (2020) with 1,404 observations.

### 2.2 Genotype filtering

A comprehensive genotype filtration was performed to ensure data quality and reliability. Initially, genotype data were extracted from the VCF file and converted into PLINK binary format. A minor allele frequency (MAF) filter was applied, retaining SNPs with an MAF greater than 0.05 to exclude rare variants. Next, linkage disequilibrium (LD) pruning was conducted to remove SNPs in high LD, using an *r* ^2^ threshold of 0.9 within a sliding window of 1,000 base pairs. The resulting dataset was recoded to a raw genotype file. Prediction accuracy was calculated as the correlation of phenotype and predicted breeding value.

### 2.3 Model implementation

ShinyGS integrates multiple GS algorithms from various packages: rrBLUP, BWGS, GBM, and DNNGP. The rrBLUP, BWGS, and GBM packages are implemented in R libraries, whereas DNNGP is called from a Python module. The rrBLUP method is from the “rrBLUP” package. It is a fast maximum-likelihood algorithm for mixed models, assuming that all markers have equal variance with small but non-zero effects (Endelman, 2011). This model estimates the marker effects from training datasets and ultimately estimates the genomic estimated breeding values (GEBVs) for the selection of candidates. BWGS is an integrated package compiling various R libraries for easy computation of (GEBV) (Charmet et al., 2020). The GBLUP, MKRKHS, RR, BRR, LASSO, EN, BL, BA, BB, BC, RF, and SVM models are included in this package. The GBM method is from the “gbm” package (Ridgeway, 2007). It mainly takes the gradient boosting (Friedman, 2001) and stochastic gradient boosting approaches (Friedman, 2002). This method is especially appropriate for mining less than clean data. DNNGP is a Python pipeline, developed based on deep neural network-based method. It can be used to predict phenotypes of plants based on multi-omics data (Wang et al., 2023).

## 3 Results and discussion

### 3.1 ShinyGS application overview

**Figure 1:**
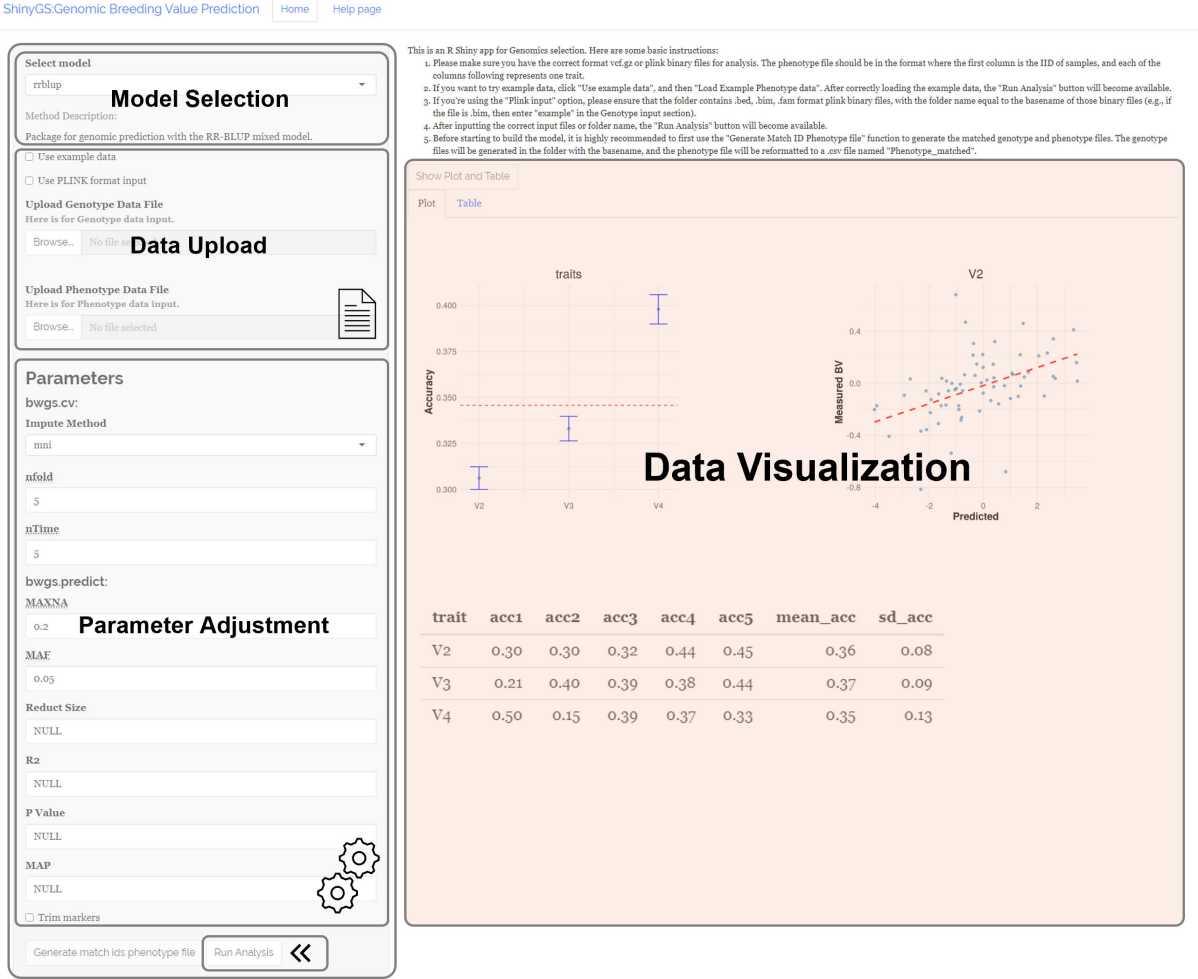
Work panel of the ShinyGS application.

ShinyGS is an R shiny application integrating a series of genetic and machine learning models for genomic selection. The application interface comprises four main sections: Model Selection, Data Upload, Parameter Adjustment, and Data Visualization. This application includes 16 genomic prediction algorithms, including rrBLUP, DNNGP, GBM, GBLUP, MKRKHS, RR, LASSO, EN, BRR, BL, BA, BB, BC, RKHS, RF, and SVM, for users to select in the “Model Selection” panel (**Figure 1**). Users can upload genotype data files in VCF format and phenotype data files in TXT format via the “Data Upload” panel. Upon uploading the correct files, a “Run Analysis” button appears. Users can adjust model parameters based on the selected genomic prediction models. After the analysis is completed, a scatterplot with predicted breeding values and raw phenotype is generated, and a table with predicted breeding values can be downloaded in the “Data Visualization” panel (**Figure 1**).

### 3.2 Demonstration of ShinyGS functionalities

In this section, we will demonstrate the functionalities using a maize diversity panel with 282 resequenced genotypes and measured days to flowering (DTF).

**Figure 2:**
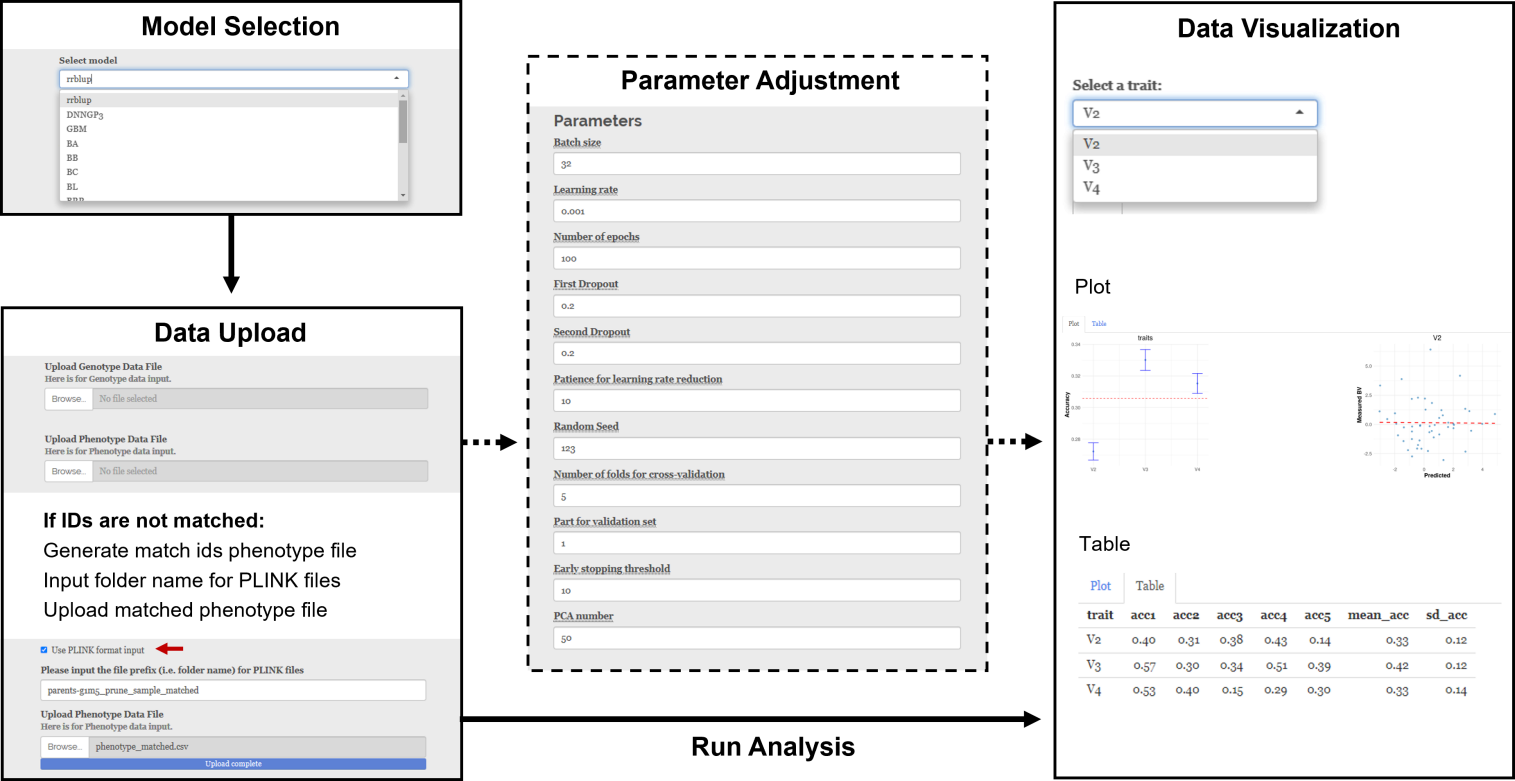
Demonstration of ShinyGS by predicting DTF in a maize diversity panel with 282 recombination intercross populations (RILs).

- Model Selection: A total of 16 models are available for selection from the drop-down tab in the “Select Model” panel (**Figure 2**).
- Parameter Adjustment: For models without any additional parameters, such as the rrBLUP model, the “Parameter Adjustment” panel does not appear when these models are selected. Otherwise, a parameter adjustment panel will show up. For example, when using the BWGS method set, users can set the imputation method, max NA, MAF, size reduction, *R* ^2^, *P*-value, and MAP. When using the DNNGP model, users can set batch size, learning rate, number of epochs, first dropout, second dropout, patience for learning rate reduction, random seed, number of folders for cross-validation, part for validation set, early stopping threshold, and number of PCA.
- Data Upload: Users can upload genotype and phenotype files in the Data Upload section. Both “.vcf” and “.vcf.gz” file formats are acceptable for genotype files. A vcf file contains genetic markers for genomic selection. For phenotype files, ShinyGS accepts both “.txt” and “.csv” formats, with IDs in the first column. Raw phenotype needs to be preprocessed accordingly before it can be pushed into our software. The phenotype file could include a header with ID and trait names. However, this is not mandatory. Input phenotypes without a header will be assigned with a header starting with a V-column number. ShinyGS links input genotype and phenotype files with IDs, so it is important to make sure that IDs in the two files are consistent. If not, users can create an ID-matched phenotype file using the “Generate match IDs phenotype file” function. Alternatively, ShinyGS also accepts genotype and phenotype in PLINK format. This can be done by checking the “Use PLINK format input” box and input the folder name with PLINK files into the genotype box.
- Run Analysis: Once the above steps are completed, a “Run Analysis” button appears.
- Results and Visualization: After the analysis is completed, a scatterplot with predicted breeding values and raw phenotype is generated, and a table with predicted breeding values can be downloaded.

### 3.3 Benchmarking model performances for a number of traits using a recombination intercross population

In this section, we benchmarked the performance of the 16 models using a maize multiple parental advanced intercross population (CUBIC) (Liu et al., 2020). This population was derived from 24 elite Chinese maize inbred lines from four divergent heterotic groups, and a total of 24 founders were crossed under a complete diallel cross-mating design (Liu et al., 2020). After selfing for more than 10 generations, a total of 1,404 inbred maize lines were obtained, genotyped, and phenotyped. Here, 42,267 single nucleotide polymorphisms (SNPs) and three traits—PH (cm), DTA (days), and EW (g)—were used. Prediction accuracy was calculated as the Pearson correlation between measured phenotype and predicted breeding values.

**Figure 3:**
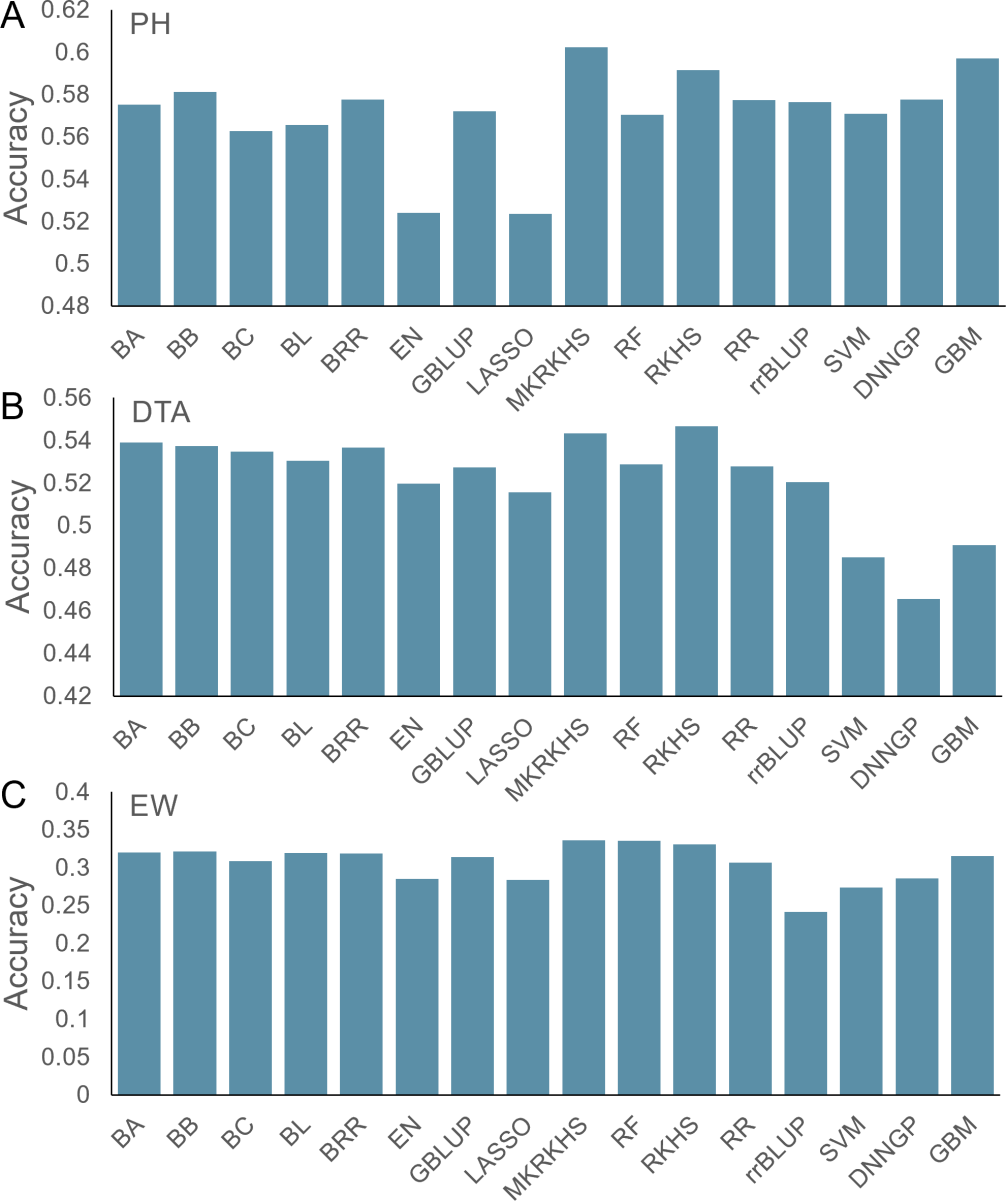
Prediction accuracy of 16 models for the maize CUBIC population. **(A)** PH, **(B)** DTA, and **(C)** EW.

For PH, the prediction accuracy varied from 0.52 to 0.60, with an average of 0.57 (**Figure 3**). The MKRKHS model displays the highest accuracy (0.60), while the LASSO model displays the lowest accuracy (0.52). For DTA, the average prediction accuracy is 0.52, and the SVM, DNNGP, and GBM models show accuracies lower than 0.5 (**Figure 3**). Due to relatively low heritability, the average accuracy for the EW dataset is 0.31 with MKRKHS yielding the highest prediction performance (**Figure 3**).

Although the prediction accuracies varied between traits and models, MKRKHS showed the highest accuracies for all three traits. We, therefore, recommend using the MKRKHS model as a first choice in intercross population in future applications.

### 3.4 Benchmarking model performances for a number of traits using a maize diversity panel

In this section, we benchmarked the performance of the 16 models using the maize Goodman diversity panel, which included 26 stiff stalk lines, 103 non-stiff stalk lines, 77 tropical/subtropical lines, 6 sweet corn lines, 9 popcorn lines, and 61 mixed lines (Kremling et al., 2018). Compared with the CUBIC population, this population is highly stratified, covering major *Zea mays* varieties across the world. There were 16,238 SNPs, and three phenotypes—DTA (days), PH (cm), and EW (g)—were used.

**Figure 4:**
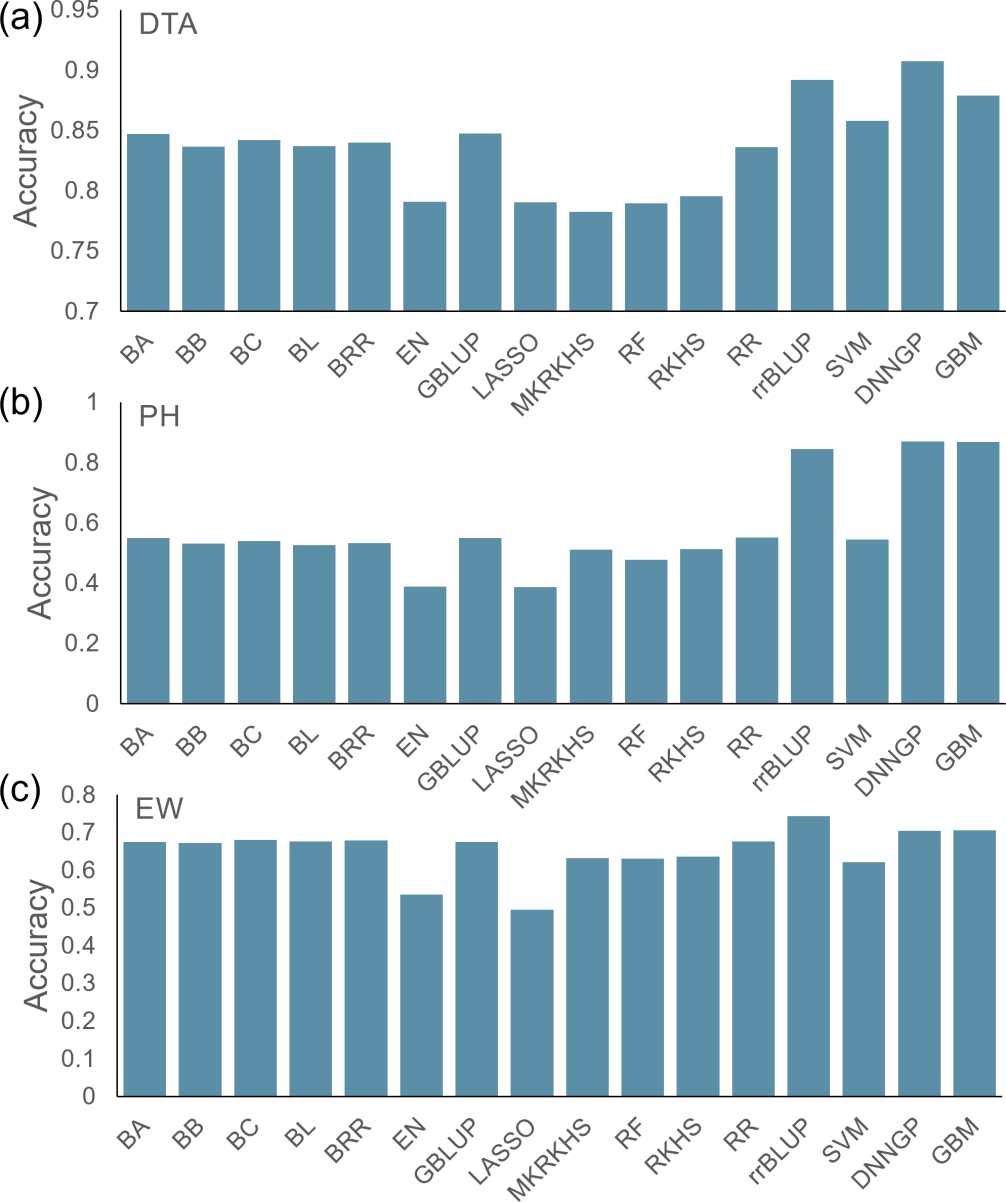
Prediction accuracy of 16 models for the maize Goodman diversity panel. **(A)** DTA, **(B)** PH, and **(C)** EW.

The average accuracies for the three phenotypes across the 16 models were 0.84, 0.55, and 0.64, respectively (**Figures 4A–C**). Compared with the other models, EN and LASSO had lower accuracy in all three tests. Although the prediction accuracies varied between traits and models, the GBM model showed the highest accuracies for all three traits. We, therefore, recommend using the GBM model as the first choice in a diversified population in future applications.

### 3.5 Comparison of computing time and memory usage

In this section, we benchmarked computing time and memory usage in relation to population size and prediction methods. To estimate how computational resource scales with population size for each method, we calculated computing time and memory consumption by downsampling the CUBIC population to 500, 800, and 1,404 individuals.

**Figure 5:**
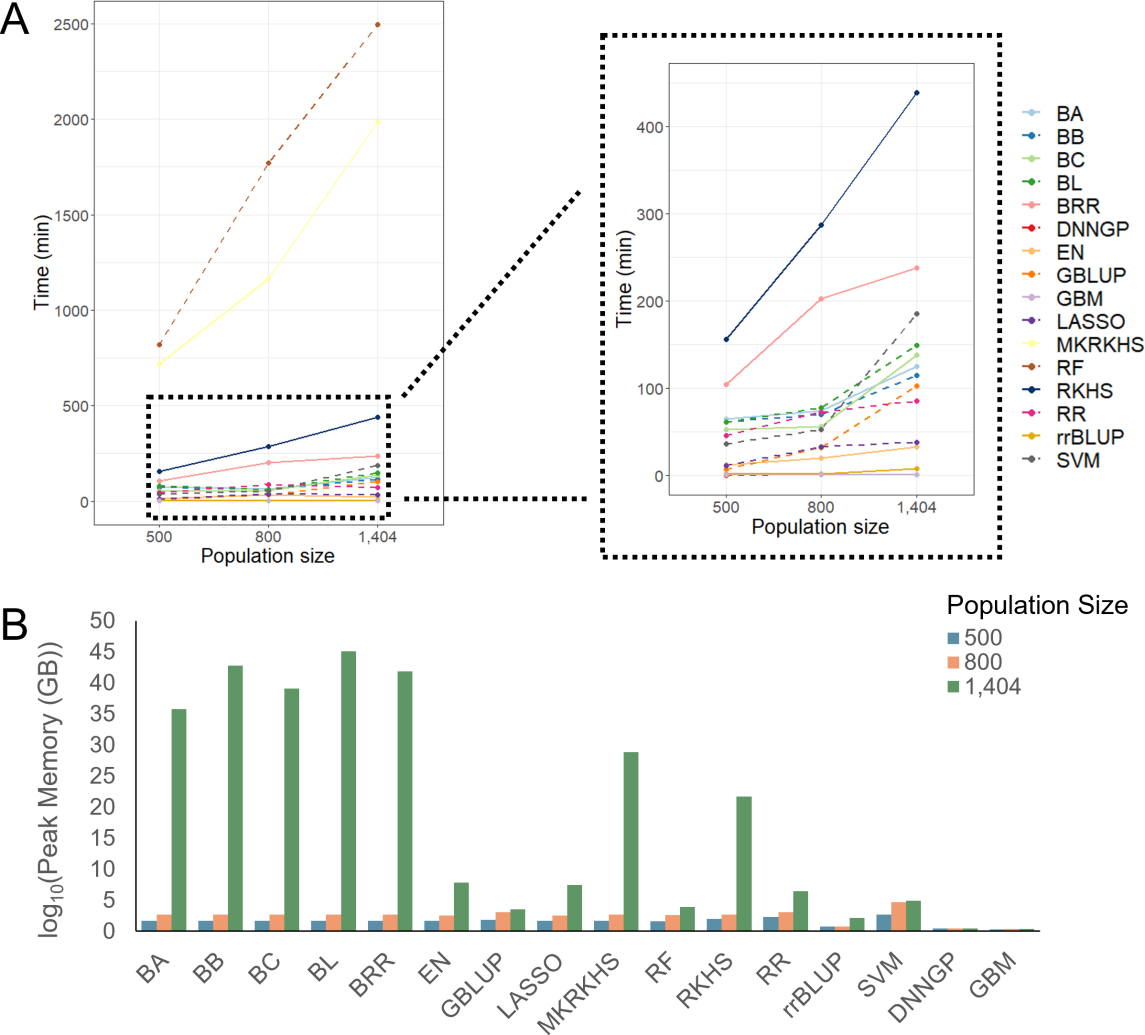
Computing time and memory usage of different models with different population sizes. **(A)** Computing time of 16 models. **(B)** Memory usage of 16 models.

Overall, most models displayed increased computing time with a larger population size. There are two models, RF and MKRKHS, that took more than 500 min, regardless of population size. The RF model took a longer computing time than the other models, and its time consumption increases linearly as the population grows (**Figure 5**).

For memory usage, most models used less than 3 GB memory at population sizes of 500 and 800 but increased sharply at a population size of 1,404. The GBLUP and SVM models used the largest amount of memory at a population size of 800. In contrast, the DNNGP and GBM models showed stable memory usage (**Figure 5**).

Overall, Bayesian methods scale poorly with sample size and cannot outperform other methods in nearly all the benchmarked traits and populations. We suggest users to leave them as the last option. Taking the prediction performance, computational time, and resources together, GBM could be the first choice as it gives satisfying performance under reasonable time especially when computational resources are limited. Under limited computational resources and time, RF and MKRKHS should be the last option as they are associated with much higher costs in time and resources with no or marginal gain in accuracy. Overall, we suggest users balance the prediction accuracy and available computational resources in their breeding applications.

## Data Availability

The original contributions presented in the study are included in the article/**Supplementary Material**. Further inquiries can be directed to the corresponding authors.
