# Supplementary material for: ShinyGS—a graphical toolkit with a serial of genetic and machine learning models for genomic selection: application, benchmarking, and recommendations
Source: Front Plant Sci. 2024 Dec 24;15:1480902. doi: 10.3389/fpls.2024.1480902 (PMC11703999; doi:10.3389/fpls.2024.1480902)
Supplement: Supplementary file 1 [file DataSheet1.pdf]

ShinyGS

A graphical toolkit with a serial of genetic and machine learning models for genomic selection

Version 1.1.3 (November 2024)

Yifei Dai et.al

TABLE OF CONTENTS

1 Introduction 3

2 Installation 4

2.1 Docker installation 4

2.2 ShinyGS installation 5

3 Start 6

3.1 Model Selection 6

3.1.1 Model Function 6

3.1.2 Parameter Adjustment 7

3.2 Input 9

3.3 ShinyGS Functions 10

4 Output Results 12

1 INTRODUCTION

ShinyGS is a graphical toolkit with a serial of genetic and machine learning models for genomic selection. It is a platform-independent software that can be run under all operating systems with a docker container for quick installation. It encompasses the mainstream Genomic Selection algorithms including rrBLUP, DNNGP, GBM and BWGS method set. BWGS method set includes GBLUP, MKRKHS, RR, BRR, LASSO, EN, BL, BA, BB, BC, RKHS, RF and SVM.

Moreover, it also has a use-friendly interface to visualize the result, which can significantly simplify genomic prediction applications for breeders.

2 INSTALLATION

2.1 DOCKER INSTALLATION

As this ShinyGS is embedded in a docker image, it is necessary to install Docker on your device. Please follow the corresponding instructions according to your operating system.

Linux OS - For Linux OS user, you can use the following command to download docker:

```
sudo apt-get update
sudo apt-get install docker-ce docker-ce-cli containerd.io
```

After installation, you can check if Docker was successfully installed with:

```
docker --version
```

If you get the docker version correctly, then you are able to continue to the next section.

Windows - For Windows users, you need to have WSL 2 on your computer before running Docker. Then you need to download Docker Desktop for your device. Here's the step-by-step procedure:

1. Ensure you have WSL 2 set up. If not, follow the Microsoft documentation to set it up: https://docs.microsoft.com/en-us/windows/wsl/install
2. Navigate to the official Docker website or use the following link: https://www.docker.com/products/docker-desktop
3. Click on the "Download for Windows" button.
4. Once downloaded, run the installer and follow the on-screen instructions.
5. After installation, you might need to restart your computer.
6. Once restarted, you can launch Docker Desktop from the Start Menu or Desktop shortcut.
7. Verify the installation by right-clicking the Docker icon in the system tray and selecting "About Docker Desktop".

Note: Ensure that your Windows version supports Docker Desktop and WSL 2. Docker typically requires Windows 10 64-bit. For older versions or if you encounter issues with WSL 2, consider using Docker Toolbox and the legacy Docker Desktop using Hyper-V. This incorporates the need for WSL 2 when using Docker Desktop for Windows with the WSL 2 backend.

After ensuring that WSL 2 and Docker Desktop are properly set up:

1. Press 'Ctrl+R' on your keyboard to open the "Run" dialog.
2. Type cmd and press 'Enter' to open the Command Prompt.
3. In the Command Prompt, type wsl and press 'Enter' to access the WSL 2 Linux environment.
4. You should now be in the Linux terminal. You can verify this by typing docker -version which will display information about the docker version. If you correctly get the version of Docker, then you are able to continue to the next section.
5. To exit the WSL 2 environment and return to the Command Prompt, simply type exit and press 'Enter'.

2.2 SHINYGS INSTALLATION

After having docker properly set up on your device, you can then simply use

```
docker pull yfd2/ags:1.1.3
```

to get the docker image of this software. Once you have accomplished this step, you can use

```
docker images
```

to check if this image is on your device.

Note: The downloading process is relatively time-consuming, please don't interfere the downloading process.

3 START

Once you have completed the installation steps, please make sure to run “docker images” and check if the docker image is on your device. After verification, you can then go to https://github.com/leyu0325/ShinyGS to download a bash script Run.sh. After downloading the bash script, move it to the file path you what to store the analysis result. This script automatically detects the file path and store your analysis results in this path.

Then navigate to that path:

```
cd [/path/to/the/bash/script]
```

and then use the following command to start the bash script

```
bash Run.sh
```

it will automatically open the ShinyGS in your browser. Here’s an example:

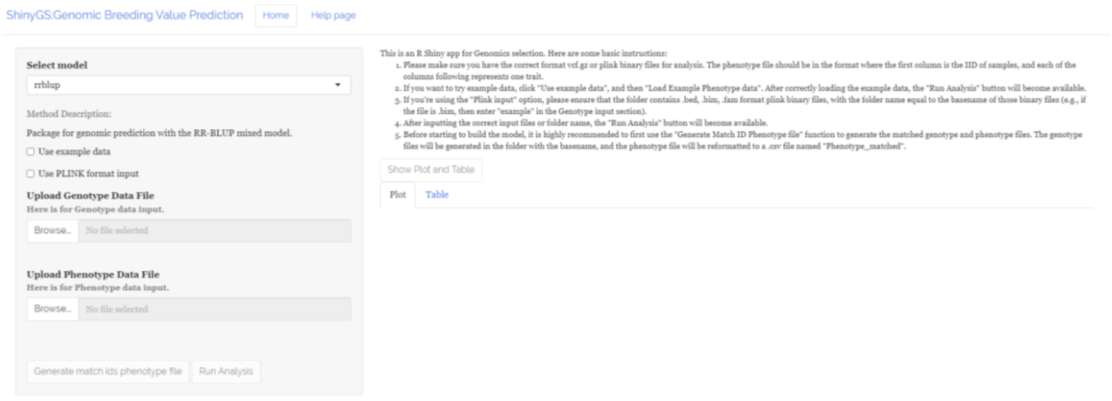

3.1 MODEL SELECTION

Once you successfully enter this page, you can start to use this ShinyGS by selecting the GS model you want to use. A total of 16 models are available for selection from the drop-down tab in the “Select model” panel.

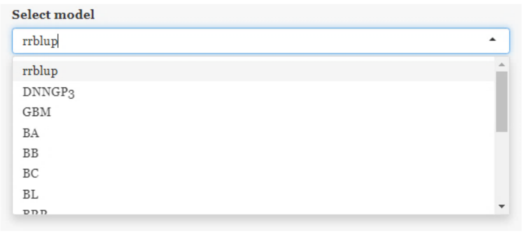

3.1.1 Model Function

Here’s a description of each model listed in the “Model Selection” part.

**rrBLUP**	Mixed linear model framework. This model estimates the marker effects from training datasets and ultimately estimates the GEBV for the selection candidates.
**DNNGP**	Deep Neural Network Genomic Prediction. For integration of multi-omics data in plants, here we only use Genomics data as the input.
**GBM**	Gradient Boosting Machine. It mainly takes the gradient boosting and stochastic gradient boosting approaches. It is called from 'gbm' package.
**GBLUP**	Performs G-BLUP using a marker-based relationship matrix, implemented through BGLR R-library. Equivalent to rrBLUP of marker effects.
**MKRKHS**	Multiple Kernel Reproducing Kernel Hilbert Space (MKRKHS) using BGLR. Based on genetic distance and a kernel function to regulate the distribution of marker effects. This method is claimed to be effective for detecting non-additive effects.
**RR**	Ridge regression, using package 'glmnet'. In theory, strictly equivalent to GBLUP.
**BRR**	Bayesian ridge regression. Same as rrBLUP, but with Bayesian resolution. Induces homogeneous shrinkage of all marker effects towards zero with Gaussian distribution.
**LASSO**	Least Absolute Shrinkage and Selection Operator is another penalized regression method which yields more shrunken estimates than RR. Run by 'glmnet' library.
**EN**	Elastic Net, which is a weighted combination of RR and LASSO, using 'glmnet' library.
**BL**	Bayesian LASSO uses an exponential prior on marker variances, leading to double exponential distribution of marker effects. Run by 'BGLR' library.
**BA**	Bayes A uses a scaled-t prior distribution of marker effects. Run by 'BGLR' library.
**BB**	Bayes B, uses a mixture of distribution with a point mass at zero and with a slab of non-zero marker effects with a scaled-t distribution. Run by 'BGLR' library.
**BC**	Bayes C is the same as Bayes B but with a slab with Gaussian distribution. Run by 'BGLR' library.
**RKHS**	Reproductive Kernel Hilbert Space using BGLR. Same as MKRKHS.
**RF**	Random forest regression, using the random Forest library. This method uses regression models on tree nodes which are rooted in bootstrapping data. It's supposed to be able to capture interactions between markers.
**SVM**	Support vector machine, run by the e1071 library. For details, see Chang, Chih-Chung and Lin, Chih-Jen: LIBSVM: a library for Support Vector Machines http://www.csie.ntu.edu.tw/~cjlin/libsvm.

3.1.2 Parameter Adjustment

For some models, there are additional parameters that you may want to adjust.

When using DNNGP model:

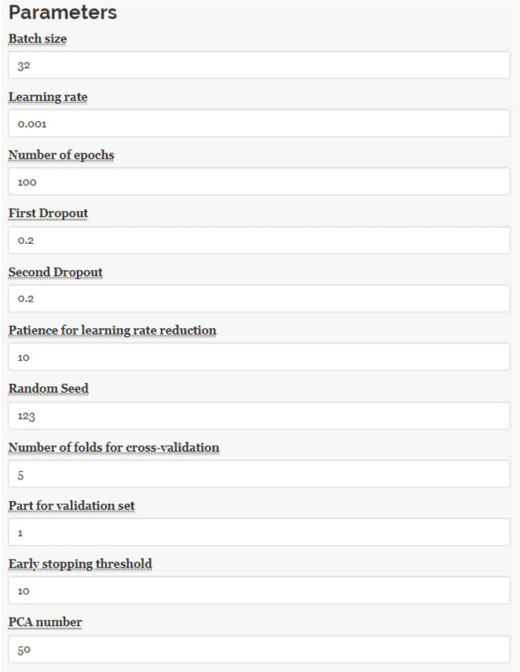

When using GBM model:

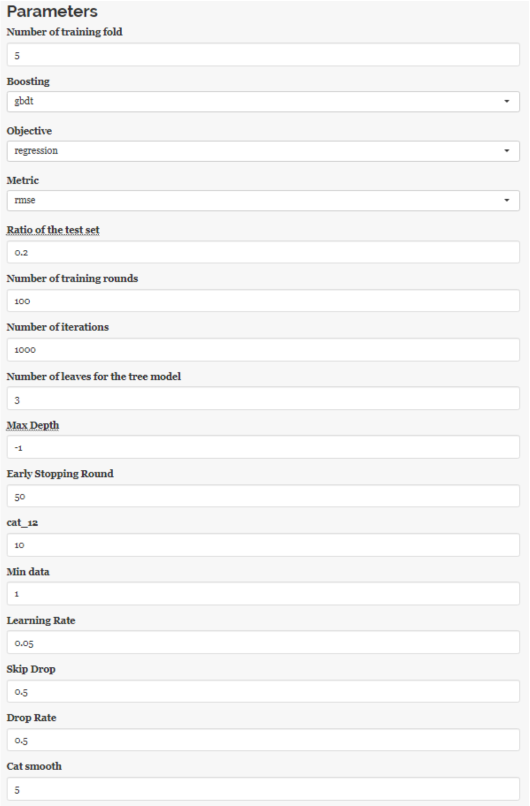

When using BWGS method set:

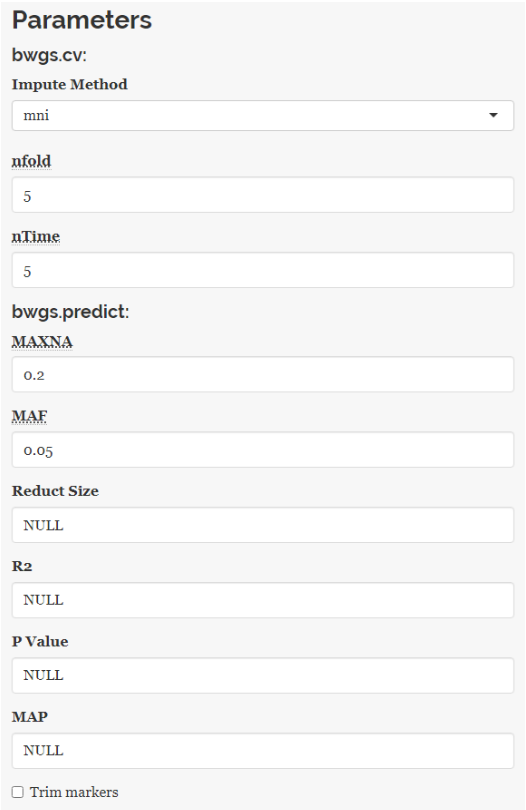

3.2 INPUT

Once you have selected and set up a model, you can then upload genotype and phenotype files in "Data Upload" section. ShinyGS uses variant ID as the bandage between genotype file and phenotype file, so make sure you have the correct version of IDs. For genotype file, either ".vcf" or ".vcf.gz" file format is acceptable. For the phenotype file, ShinyGS accepts both ".txt" and ".csv" format, with IDs in the first column. Genotype file should contain the markers you would like to be used to do the Genomic Selection.

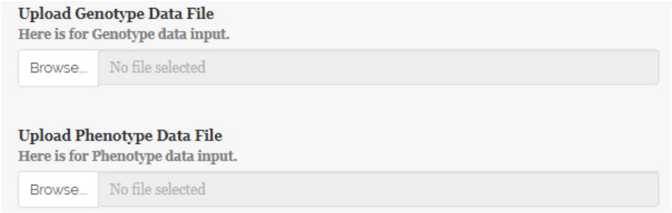

Phenotype File Format - Raw phenotype needs to be preprocessed accordingly before pushed in to our software. The phenotype file could include a header with id and trait names. However, this is not mandatory. Input phenotypes without header will be assigned with a header starting with V-column number. There is no limit on the maximum number of characters. The first column of the phenotype file should contain SampleIDs that match the IIDs in the genotype file. The matching process can be facilitated by ShinyGS using the

"Generate matching IDs phenotype file" function. This function generates a CSV file with matched SampleIDs and genotype IIDs, ready for further analysis. The traits (phenotypes) for these samples are organized from the second column to the last column, with each column representing a distinct trait or phenotype. Any missing values within these columns will be treated as targets, with corresponding predictions generated based on the trained model and the available phenotypes.

SampleID	Trait1	Trait2	Trait3
Yu796	74.929	150.881	74.268
Wf9	72.584	167.717	75.022
W182B	64.676	.	49.271
W153R	71.453	101.599	49.881
W117Ht	67.092	109.001	48.839
VaW6	.	105.409	48.153
Va99	74.135	156.995	61.204
Va85	74.82	161.486	75.071
Va59	72.433	142.94	65.569
Va35	74.988	141.835	66.107

Each column is a phenotype/trait

Treated as target, based on the model trained to predict these values

Note: Remember the ID in phenotype file should correspond to that of genotype file. If not, you can use function "Generate match ids phenotype files". If the ID matches, then you will find the output in the corresponding folder, and those NA in your trait columns will automatically be considered and predicted.

3.3 SHINYGS FUNCTIONS

Generate match ids phenotype file - This function is mainly about creating an ID matched phenotype file. You can find the new matched phenotype file in /mount/folder_name/. Remember to click the "Use PLINK format input" checkbox and input the folder name to the genotype box. Then you can upload matched IDs phenotype file.

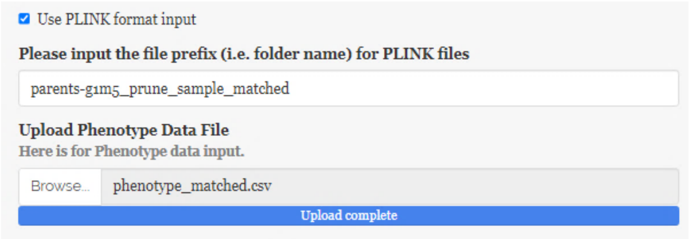

You can also use this function to check if your phenotype data's ID matches that of genotype data. There are a couple of situations here:

The length of IDs is not the same - This would raise an error. If the length is not matching, then this process will not proceed, you need to check and either edit your VCF file or your phenotype file, make sure you're clear about the relationship between your phenotype data and your genotype data, and assign them the correct version of IDs.

The length is the same and the ids are partially overlapped - This would not raise an error. This means that some of your ids overlapped but not all of them, which might be the situation you expected (or not). In this case, once you used the “Generate match ids phenotype file”, then the software will generate the overlapped phenotype file with the suffix “matched”, you can then find the output file in the output folder mentioned in the Output section.

The length is the same and the ids are the same - This would not raise an error. This means that your original phenotype file is the right version, you don’t need to edit anything. As mentioned above, the “matched” file would still be generated in the output folder. You can use your original phenotype file or the matched one to do the following analysis.

Run analysis - This is the button for running the process, make sure you have all the requirements fulfilled.

4 OUTPUT RESULTS

After the analysis is completed, an error bar plot with prediction accuracy of each trait, a scatterplot with predicted breeding values and raw phenotype are generated, and a table with predicted breeding values can be downloaded.

Prediction accuracy of each trait:

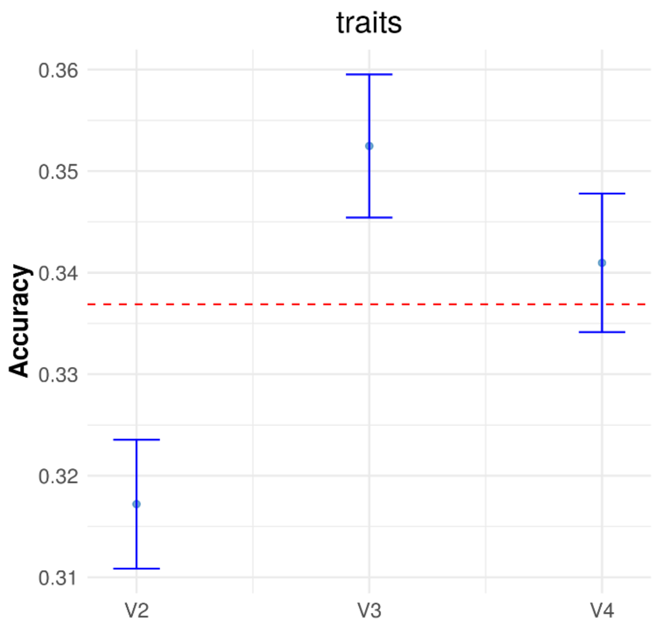

Predicted breeding values and raw phenotype:

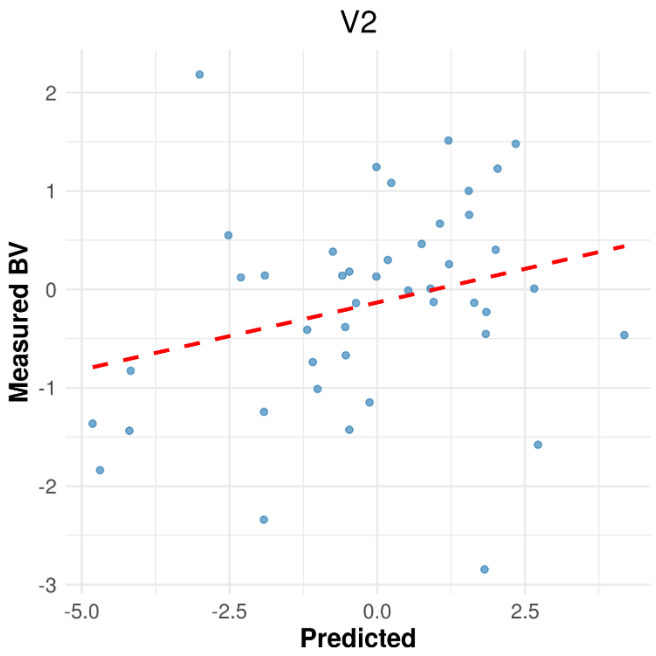

Predicted breeding values:

trait	acc1	acc2	acc3	acc4	acc5	mean_acc	sd_acc
V2	0.32	0.34	0.24	0.42	0.38	0.34	0.07
V3	0.25	0.40	0.48	0.28	0.31	0.34	0.09
V4	0.52	0.38	0.52	0.28	0.25	0.39	0.13

You can find all output results in the directory where the bash script Run.sh is stored. Each trait will have a folder to store the generated plots and tables.
